# Therapeutic Implications of PPAR*γ* in Human Osteosarcoma

**DOI:** 10.1155/2010/956427

**Published:** 2010-02-16

**Authors:** Eric R. Wagner, Bai-Cheng He, Liang Chen, Guo-Wei Zuo, Wenli Zhang, Qiong Shi, Qing Luo, Xiaoji Luo, Bo Liu, Jinyong Luo, Farbod Rastegar, Connie J. He, Yawen Hu, Barrett Boody, Hue H. Luu, Tong-Chuan He, Zhong-Liang Deng, Rex C. Haydon

**Affiliations:** ^1^Molecular Oncology Laboratory, Department of Surgery, The University of Chicago Medical Center, Chicago, IL 60637, USA; ^2^Key Laboratory of Diagnostic Medicine, Chongqing Medical University, Chinese Ministry of Education, Chongqing 400016, China; ^3^Department of Orthopaedics, West China Hospital, Sichuan University, Sichuan 610041, China

## Abstract

Osteosarcoma (OS) is the most common nonhematologic malignancy of bone in children and adults. Although dysregulation of tumor suppressor genes and oncogenes, such as Rb, p53, and the genes critical to cell cycle control, genetic stability, and apoptosis have been identified in OS, consensus genetic changes that lead to OS development are poorly understood. Disruption of the osteogenic differentiation pathway may be at least in part responsible for OS tumorigenesis. Current OS management involves chemotherapy and surgery. Peroxisome proliferator-activated receptor (PPAR) agonists and/or retinoids can inhibit OS proliferation and induce apoptosis and may inhibit OS growth by promoting osteoblastic terminal differentiation. Thus, safe and effective PPAR agonists and/or retinoid derivatives can be then used as adjuvant therapeutic drugs for OS therapy. Furthermore, these agents have the potential to be used as chemopreventive agents for the OS patients who undergo the resection of the primary bone tumors in order to prevent local recurrence and/or distal pulmonary metastasis.

## 1. Introduction

Osteosarcoma (OS) is the most common nonhematologic malignant tumor of bone in adults and children, with the peak incidence in early childhood [1, 2]. It is associated with a poor prognosis due to its high grade at presentation, resistance to chemotherapy, and propensity to metastasize to the lungs [3, 4]. Furthermore, while 80% of OS patients are believed to have micrometastatic disease, only 10%–15% present as radiographically detectable lesions [5, 6]. Herein lies the challenge in identifying the 20% OS patients without micrometastases and modifying medical and surgical management accordingly. Genetic markers associated with metastatic disease could potentially spare those patients that need for chemotherapeutic agents, such as adriamycin, cisplatin, or methotrexate, and experience its severe toxicities ranging from cardiotoxicity to renal dysfunction.

**Figure 1:**
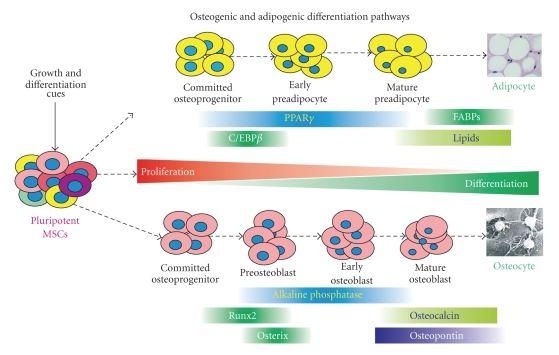
Osteogenic and adipogenic differentiation pathways in mesenchymal stem cells (MSCs). MSCs are pluripotent progenitor cells that are able to differentiate into several lineages, including osteogenic and adipogenic lineages, upon the stimulation with distinct growth and differentiation cues. The lineage-specific differentiation is a multiple-stage and well-coordinated process regulated by master regulators, such as PPAR*γ* and C/EBP*β* for adipogenesis and Runx2 and Osterix for osteogenesis. Osteogenic differentiation can be staged by measuring alkaline phosphatase (early marker) and osteocalcin and osteopontin (late markers). Expression of FABPs and production of lipids are indicators of terminal adipogenic differentiation.

**Figure 2:**
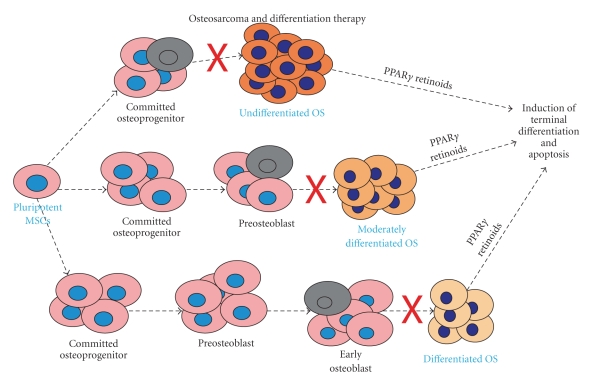
Osteosarcoma (OS) development and nuclear receptor agonist-mediated differentiation therapy. OS can be regarded as a differentiation disease, which is caused by disruptions of the terminal osteogenic differentiation. The stage and nature of differentiation defects may determine the aggressiveness of OS tumors. PPAR*γ* agonists and/or retinoids have been shown to inhibit OS proliferation, induce apoptosis, and promote osteogenic differentiation. Thus, these agents can be used as differentiation therapy, in combination with conventional chemotherapy, for OS treatment.

It has been shown that OS cells are similar to undifferentiated osteoblasts, and increasing evidence suggests that osteogenic differentiation defects may be responsible for OS tumorigenesis [2, 7–10]. Osteoblasts are derived from mesenchymal stem cells (MSCs), and osteoblastic differentiation is a tightly regulated process by numerous growth and differentiation factors, such as bone morphogenetic proteins (BMPs) and Wnts [2] (Figure 1). It is conceivable that any disruption of osteogenic terminal differentiation may result in the development of OS. The aggressiveness of OS may depend on the stage of disruption; that is, more aggressive OS phenotypes may be developed from mutant early osteoblast progenitors, whereas benign tumors may arise from disruptions of late stage osteoblasts [2, 7] (Figure 2).

Current cancer therapies primarily target the proliferative compartment of tumor cells. While effective in initial treatment, these strategies are often nullified by subsequent drug resistance. An attractive alternative is to overcome the uncontrolled cell proliferation through promoting terminal differentiation [8, 9, 11–13]. One possibility deals with the use of agonists or antagonists of the nuclear receptor superfamily, including vitamin D3, thyroid hormone, glucocorticoids, sex hormones, retinoids, and orphan receptors [14–18]. One interesting subgroup of the nuclear receptor superfamily is peroxisome proliferator-activated receptors (PPARs), which play a role in both promoting tumorigenesis and inducing terminal differentiation and apoptosis. Antitumor activity of PPAR*γ* agonists has been shown in tumor cells derived from liposarcoma, colon cancer, breast cancer, leukemia, gastric cancer, nonsmall cell lung cancer, and prostate cancer [19–31]. Furthermore, PPAR*γ* agonists have the potential to induce terminal differentiation in osteosarcoma cells [2, 9, 32]. In this review, we focus on the functional role of PPARs and their cross-talk with other nuclear receptors in osteogenic differentiation and tumorigenesis and the potential use of PPAR*γ* agonists as chemotherapeutic and/or chemopreventive agents for human OS.

## 2. PPARs and Their Ligands

PPARs are ligand-activated transcription factors that achieve functionality after forming a heterodimer with the 9-cis retinoid X receptor (RXR). The subsequent transcriptional activity is modulated by nuclear receptor coactivators and corepressors [33], such as C/EBP PGC1 (*α*/*β*), PRIP, N-Cor, SRC-1, p300, Hsp-72, and PBP [34–39]. The ligands include the synthetic thiazolinediones and fibrates and endogenous fatty acids and eicosanoids [40, 41]. Upon ligand binding and heterodimerization with RXR, PPARs recognize PPAR response elements (PPREs) containing the direct repeat sequence (DR-1) AGGTCA [33, 34]. PPARs can also repress gene transcription through interfering with NF*κ*B, STAT, and AP-1 signaling pathways [41–43]. Three subtypes of PPARs have been identified, PPAR*α*, PPAR*β*, and PPAR*γ* [16, 44]. PPAR*α* is found in liver, brown fat, kidney, heart, and skeletal muscle. PPAR*β* (also known as PPAR*δ*) is expressed in the gut, kidney, brain, skeletal muscle, and heart [34, 45]. PPAR*γ* is expressed primarily in adipose tissue and to a lesser extent in large intestine, kidney, prostate, cartilage, osteoblasts, epithelial cells, and monocytes [46].

PPARs play a role in diabetes, atherosclerosis, obesity, the inflammatory response, and cancer [47]. PPARs regulate the expression of many genes associated with lipid storage, *β*-oxidation of fatty acids, terminal differentiation of preadipocytes, and modulation of the body's response to insulin and glucocorticoids [16, 47–50]. PPAR*α* functions primarily in lipid catabolism, lipoprotein metabolism, and inflammation, as its expression increases with stress, glucocorticoid expression, exercise, and fasting [47, 51, 52]. PPAR*α* knockouts develop normally, however, exhibit hepatomegaly from lipid accumulation and liver tumors, impaired wound healing, prolonged inflammatory responses, and increased adipose tissue [53]. PPAR*β* has a relatively diverse range of functions, including *β*-oxidation of fats, tumorigenesis [54], vascular integrity [55], and bone metabolism [16]. PPAR*β* knockouts have fatal placental defects secondary to abnormal vascular development, and those that survive become small, but healthy adults [53]. PPAR*γ* affects the storage of fatty acids in adipose tissue, while also opposing TNF*α* and IL-6 production in inflammatory responses and insulin sensitization [47, 49, 50, 56, 57]. PPAR*γ* knockouts are embryo-lethal as the placenta fails to implant and develop properly, and those that survive display severe metabolic, intestinal, hepatic, and adipogenic abnormalities [53].

## 3. Formation of PPAR and RXR Heterodimeric Receptor Complexes

**Figure 3:**
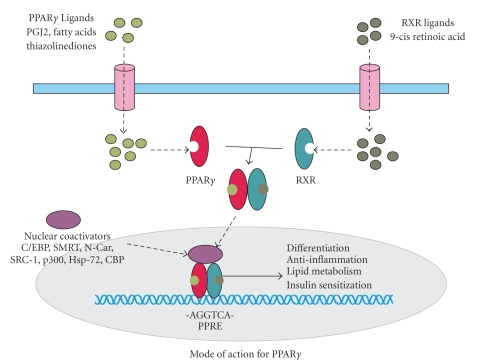
Current mode of action for PPAR*γ*. PPAR*γ* and RXR form a heterodimer, which is activated by the respective ligands. The activated PPAR*γ*/RXR heterodimer will be translocated into nucleus and regulates downstream target genes in concert with nuclear receptor coactivators.

PPARs consist of 4 domains, AB, C, D, and E. The AB domain function has not been clearly elucidated. The C domain represents the DNA binding domain (DBD), whereas the D domain contains the DBD carboxyl group along with the hinge connecting the C and E domains. The E domain (LBD) has a variety of functions, including ligand binding, hormone transactivation, and dimerization interface [16, 34, 45]. The three-dimensional structure of the LBD domain is very well conserved amongst the thyroid hormone receptor (TR*α*1), retinoic acid receptor (RAR), and retinoid X receptor (RXR) [58–60]. RXR is a promiscuous receptor and able to heterodimerize with RAR, PPAR, VDR, TR, and orphan receptors. This enables the competition among multiple hormones and other ligands to exert a variety of effects within the same tissue. It has been demonstrated that high concentrations of thyroid hormone inhibit the ability of PPAR*γ* to heterodimerize with RXR and, therefore, blocks transcriptional activation (Figure 3) [61]. Transrepression occurs through sequestration of the coactivators CBP and SRC-1 by the PPAR/RXR heterodimers, preventing their utilization in other signaling pathways [62].

A unique association with PPAR and RXR is the direct repeat responsive elements (DR) and the PPAR response elements (PPRE) associated with the heterodimer. DR1 is a repetition of 2 core motifs consisting of AGGTCA spaced apart from one another by one nucleotide in the promoter of multiple target genes (Figure 3) [63]. These motifs are recognized by two zinc finger-like motifs in the DBD region of PPAR. These PPREs produced by the PPAR : RXR heterodimer are different from those recognized by the vitamin D receptor (DR3), thyroid hormone receptor (DR4), and retinoic acid receptor (DR2, DR5) [34, 64, 65]. The importance of PPAR*γ* : RXR interaction is seen in Familial Partial Lipodystrophy, an autosomal dominant condition associated with metabolic syndrome, characterized by dyslipidemia, abnormal adipose tissue distribution, and a number of metabolic abnormalities [66]. This syndrome is associated with multiple missense and nonsense mutations of PPAR*γ* that affect its ability to dimerize with RXR and bind coactivators.

## 4. Diverse Functions of PPAR*γ*

PPAR*γ* is of particular interest because of its roles in adipogenesis, atherosclerosis, inflammation, proliferation, differentiation, and apoptosis [67]. The PPAR*γ* gene contains 3 promoters, producing 2 different proteins, PPAR*γ*1 and PPAR*γ*2, which likely contributes to this diverse range of functions [55]. PPAR*γ* expression in vascular endothelium and smooth muscle cells leads to an inhibition of MMPs, downregulation of Angiotensin II type 1 receptor, and alteration of macrophage invasion [68–71]. In inflammatory responses, its strongest association occurs with the ligand prostaglandin J2 (PGJ2) [16, 42, 72]. This eicosanoid metabolite binds directly to PPAR*γ*, leading to its activation in the inflammatory response cascade. Furthermore, the insulin sensitizing effects of PPAR*γ* are demonstrated using the synthetic antidiabetic therapy thiazolinediones [42, 72, 73]. Thus, the promiscuity of its ligand binding and a variety of associated nuclear proteins enables the diversity of PPAR*γ* functions in many tissues.

For adipogenesis, PPAR*γ* binds to fatty acids and their derivatives, such as linoleic acid and docosahaxaenoic acid (DHA) [34, 43]. These ligands activate PPAR*γ*, stimulating preadipocytes to differentiate and initiate the steps required in lipid storage. PPAR*γ* effects are carried out through target genes, such as aP2, lipoprotein lipase (LPL), acyl-Coa synthetase (ACS), and CD36 [74, 75]. Overexpression of PPAR*γ* in fibroblasts initiates the adipogenic cascade, while PPAR*γ* knockout mice are unable to form adipocytes or adipose tissue [48, 49, 76]. In humans, an activating mutation in PPAR*γ* leads to increased adipogenesis and obesity [77]. Another mutation decreasing PPAR*γ* activity results in lower body mass index [78, 79].

Adipose tissue targets of PPAR*γ* include LPL and fatty acid transporter FATP in stimulating fatty acid uptake, malic enzyme in NADPH synthesis for lipogenesis, phosphoenolpyruvate carboxykinase in gluconeogenesis, and ACS in FA esterification [16, 80–82]. PPAR*γ* promotes adipocyte differentiation of MSCs through various signaling pathways [83–85]. MSCs can differentiate into either osteogenic or adipogenic lineage, depending on the differentiation cues (Figure 1). It has been reported that osteogenesis and its signaling cascade are inhibited by PPAR*γ* activation [86–89]. However, the extent of PPAR*γ*-associated adipogenic stimulation or osteogenic inhibition depends on the nature of the ligands [90]. Such ligand-dependent regulatory functions of differentiation may explain the shift away from osteogenesis in the aging process, due to an increase in the number of bone marrow adipocytes, oxidized LDL metabolites, and fatty acids metabolites.

## 5. Role of PPAR*γ* in Osteogenesis and Adipogenesis of MSCs

Osteogenesis and adipogenesis appear to originate from the same progenitor bone marrow mesenchymal stem cells (MSCs) [91–93]. MSC differentiation into osteoblast or preadipocytes occurs through a complex regulation of events [91–94] (Figure 1). Bone morphogenetic proteins (BMPs) play an important role in this differentiation process and subsequent bone formation [2, 93, 95–101]. BMP-2, BMP-6, and BMP-9 regulate targets associated with osteoblast differentiation, while BMP-2, BMP-4, and BMP-7 appear to be associated with preadipocyte differentiation [93, 101–110]. Mice with BMP2-regulated Schnurri-2 knockout showed a reduction in white fat mass [111].

PPAR*γ* can stimulate adipocyte differentiation of MSCs. PPAR^+^ blasts (progenitor cells) have been noted in hematopoeitic lineages, and PPAR*γ* is able to induce terminal differentiation in monocytes and adipocytes [48, 50, 112, 113]. Overexpression of PPAR*γ* in fibroblastic cells initiates the adipogenic cascade, while PPAR*γ* knockout mice were unable to form adipocytes or adipose tissue [48, 49, 76]. In humans, an activating mutation in PPAR*γ* leads to increased adipogenesis and obesity, while inactivating mutation results in a lower body mass [77–79]. To demonstrate its importance, no factor has been shown to be able to induce adipogenesis in the absence of PPAR*γ* and almost all pathways involved in adipogenesis involve regulation of PPAR*γ* [114].

Furthermore, in a review by Giaginis et al. PPAR*γ* agonists were found to have a remarkable role regulating bone turnover [115]. However, while PPAR*γ* seems to shift the differentiation pathway away from osteoblastogenesis and towards osteoclastogenesis, this is not always the case. Giaginis et al. reviewed studies focusing on both the synthetic and natural PPAR*γ* ligand effects on osteoblast and osteoclast formation, as well as apoptosis and overall bone formation. They found divergent results, as it appears there are other factors that contribute to bone turnover regulated by PPAR*γ*. For example, since natural ligands are found in both the diet and the inflammatory cascade, perhaps these processes determine the final outcome of PPAR*γ*-regulated bone turnover. Its effect in clinical studies poses a similar paradox, while some patients receiving synthetic PPAR*γ* agonists for Diabetes Mellitus type II experienced bone loss, others were noted to have a decrease in bone resorption markers [115].

PPAR*γ* plays an intriguing role in both adipogenesis and osteogenesis. Earlier reports indicate that homozygous PPAR*γ* deficient progenitor cells spontaneously differentiate into osteoblasts via increased osteoblastogenic factors *in vitro*, and heterozygous PPAR*γ* deficiency results in increased *in vivo* bone formation [87]. However, recent studies have demonstrated that osteogenic BMPs can effectively induce adipogenic differentiation [101, 116, 117]. PPAR*γ* has been shown to be significantly upregulated by osteogenic BMPs [101, 118]. Overexpression of PPAR*γ*2 promotes the osteogenic BMP-induced osteogenesis and adipogenesis [101]. Silencing PPAR*γ*2 expression leads to an inhibition of adipogenic differentiation as well as stimulation of osteogenic differentiation and osteoid matrix mineralization [101]. However, it remains to be elucidated how BMP-induced MSC differentiation into osteogenesis and adipogenesis diverges.

The regulation underlying these effects could be secondary to the nuclear competition between PPAR*γ* and other members of its nuclear receptor superfamily. Regulation of the osteogenic promoter, osteocalcin, by glucocorticoids, vitamin D, and thyroid hormone, occurs through the same nuclear pathway as PPAR*γ* [119–121]. In addition, PPAR*γ* activation by fatty acids and their derivatives might lead to a slowing of osteoblast differentiation, which would explain the tendency to shift to adipogenesis. These findings are intriguing as recent studies have indicated that aging activates adipogenesis and suppresses osteogenesis, possibly through the increased availability of these fatty acids and a decrease in metabolic production of many nuclear hormones. These would shift the signaling towards PPAR*γ*, which might explain part of the mechanism underlying osteoporosis [122, 123]. 

### 5.1. Side Effects of PPAR*γ* Ligands

In the treatment of Diabetes Mellitus (DM) type II with synthetic PPAR*γ* ligands, the most common side effects observed have been headaches, gastrointestinal symptoms (nausea, diarrhea), and susceptibility to infections. Troglitazone has been withdrawn from the market secondary to its hepatic toxicity; however, this appears to be drug specific and not universal amongst PPAR agonists.

In a review by Mudaliar and Henry about the clinical use of glitazones, side effects include edema, weight gain, and mild drops in hematocrit. Rarely, increases in liver enzymes are observed. These synthetic PPAR*γ* agonists induce the cytochrome P450 isoform CYP3A4 in the liver, affecting the metabolism of many other drugs. These drugs have been shown to increase plasma volume, thus, leading to edema and a dilutional drop in hematocrit. While the mechanism has not been elicited, PPAR*γ* agonists antagonize the vasoconstriction induced by hyperinsulinemia, by sensitizing cells to the effects of insulin. Therefore, they relax vascular smooth muscles and decrease peripheral blood pressure. While there is no mention of increased peripheral adipose tissue, the propensity of PPAR*γ* to induce lipid storage might underlie the observed weight gain [124].

Theoretically, a shift away from osteogenesis and towards adipogenesis might also promote osteoporosis and increase fracture risk. This has been demonstrated in a prospective study of over 80,000 patients being treated for DM type II [125]. Furthermore, competition for the RXR heterodimer might decrease the effects of other nuclear receptors in the superfamily, having a variety of effects on many different tissues. While recently there was a report suggesting increased fracture risk in patients receiving PPAR*γ* agonists, there has been relatively little other evidence supporting any of these notions [122].

## 6. Molecular Biology of Osteosarcoma

The molecular pathogenesis underlying OS development is poorly understood. OS is associated with aberrations in p53 and Rb expression [1, 2, 126–128]. Other genetic alterations associated with OS development include p16^INK4a^, c-Myc, Fos-Jun, MDM2, CDK4, and cyclin D [1, 2, 126]. Altered cell signaling pathways in OS include Wnt, sonic hedgehog, TGF*β*/BMP families, and IGF2 [1, 2, 126]. Mutations in DNA helicase increase OS risk and MMP expression leads to a more aggressive OS tumor, while lack of telomerase activity is associated with a favorable prognosis [129–131]. The identifiable risks associated with OS include exposure to the FBJ or SV40 virus, beryllium oxide chemical, and radiation [132–134]. 

Disruption of the osteogenic differentiation pathway from MSCs is thought at least in part to be responsible for OS tumorigenesis [2, 7, 9, 10] (Figure 2). By preventing the differentiation of MSCs, the proliferative capability of preosteoblasts increases the risk for malignant transformation. It has been well established that early progenitor cells have similar characteristics to a variety of tumor cells. For example, progenitor cells of the hematopoietic system share similar leukocyte receptors to leukemic cells. Furthermore, tumor cells share many of the antiapoptotic and self renewal machinery with stem cells [135]. It has been proposed that a small subset of cancer cells, known as cancer stem cells, act in a similar manner to adult stem cells, with proliferative and regenerative capabilities that enable tumors to survive and grow [135]. In colon cancer cells, preneoplasia and neoplasia have the same nuclear morphotypes that 5–7-week-old fetal gut stem cells possess but are not found in the adult colonic crypt cells [136]. Osteosarcoma cells display similar characteristics to undifferentiated osteoblasts [7–10]. In OS cell lines, early osteogenic markers, such as CTGF, are high while late markers such as Runx2, Alkaline Phosphatase, Osteopontin, and Osteocalcin are low [7].

Therefore, investigation into the induction of terminal differentiation in cells with such differentiation defects has increased in recent years. A similar process has been reported in Ewing's Sarcoma, in which silencing the EWS/FLI-1 in Ewing's sarcoma cells leads to their recovery of MSC capability to differentiate into osteogenic lineages [137]. Accordingly, overexpression of this oncogene causes MSCs to remain in an undifferentiated state and promotes tumor growth [138]. Rb mutations occur in many OS tumors [1, 2, 126–128]. Rb coactivates the osteoblast differentiating agent Runx2 and loss of function of Rb stalls terminal osteoblast differentiation [139]. Furthermore, it has been recently shown that OS cells are refractory to BMP-induced osteogenic differentiation, whereas osteogenic BMPs promote OS growth *in vivo*, which can be overcome by introducing key osteogenic regulator Runx2 [7]. These findings suggest that the late stages of osteogenic differentiation may be preserved [7, 8]. Therefore, anti-OS therapies may be developed by promoting osteogenic terminal differentiation [2, 7]. Differentiation agents would add another dimension to the current chemotherapeutic cocktails focusing on the inhibition of the cell cycle.

### 6.1. Molecular Biology Relating to the Differentiation Status in Tumors

It appears that the differentiation status not only is responsible for the development of OS but also may predict its malignant potential. From the early 1970s when the idea of differentiation was first proposed, to more recently when differentiation agents are used for certain cancer phenotypes, it has been observed that this process is associated with many morphological changes in the respective cells. These changes leading to a well-differentiated cell include repression of responsiveness to growth factors, withdrawal from the cell cycle into a state of quiescence, and a decreased ability to re-initiate proliferation [140]. For example, as adipocytes differentiate, they progressively become less responsive to mitogenic growth factors MIX and PDGF, eventually repressing the expression of proto-oncogenes c-jun and junB [141, 142]. The more differentiated the adipocyte, the less responsive it is to growth factors. Terminal adipocyte differentiation is accompanied by expression of proteins that repress RNA expression, along with induction of p21, leading to irreversible loss of proliferative potential [140, 143]. When breast cancer's estrogen receptors were first discovered and evaluated, the notion was proposed that the cancers with estrogen receptors represent a well-differentiated class of tumors that undergo clonal evolution and eventually lose their receptor status when they become poorly differentiated [144]. Another example occurs when Simian Virus 40 large T antigen transforms cells to increase their responsiveness to growth factors and become undifferentiated [145].

The fundamental idea behind differentiation therapy for tumors is that by inducing terminal differentiation, the tumor cells lose their proliferative phenotypes. Differentiation causes cells to lose their proliferative potential and repress their responsiveness to growth factors, while at the same time possibly increasing their susceptibility to apoptosis, induction of tumor suppressors, repression of oncogenes, inhibition of angiogenesis, and induction of cytotoxic agents. As cells become more differentiated, these changes make them less aggressive and more responsive to other chemotherapeutic agents.

### 6.2. Clinical Examples of Therapeutic Success by Induction of Terminal Differentiation

Inhibition of tumor growth through differentiation therapy has been demonstrated in clinical cases of hematologic and breast tumors. Induction of terminal differentiation was first shown to be successful in treating AML with low-dosage araC [146]. Recently, there have been many more therapeutic interventions that have focused on overcoming the uncontrolled cell proliferation through terminal differentiation [8, 9, 11–13]. One possibility deals with the nuclear receptor superfamily associated with vitamin D3, thyroid hormone, glucocorticoids, sex hormones, retinoids, and orphan receptors [14–17]. Treatment focusing on counteracting hormone dependent activation of these nuclear receptors is seen in therapies such as tamoxifen for breast cancer [18]. In this and other examples, regulation of the nuclear receptor leads to differentiation, causing the cells to lose their proliferative properties and antiapoptotic tendencies.

## 7. Role of PPARs in Tumorigenesis and Differentiation

PPARs play an important role in tumorigenesis and differentiation (Table 1). PPAR*α* is responsible for hepatocarcinogenic effects in rodents [147]. PPAR*β* was identified as a downstream target of the APC/*β*-catenin pathway, associated with human colon tumors [54]. PPAR*β* has been shown as a target for nonsteroidal anti-inflammatory drug- (NSAID-) induced chemopreventive effects in colon cancer [45, 54]. High dose of NSAIDs, such as sulindac and indomethacin, displays chemopreventive effects in the familial adenomatous polyposis mouse model. It downregulates Cox-2 expression in humans, leading to a decrease in intestinal polyps, inhibition of cell cycle progression, and induction of apoptosis in colorectal tumor cells [45, 148, 149]. NSAIDs can disrupt the ability of PPAR*β* to bind to its peroxisome proliferator response elements (PPREs) *in vitro*, while PPAR*β* overexpression was able to rescue NSAID induced apoptosis in colon tumor cells [45]. These findings may explain the correlation between dietary fat consumption and colon cancer incidence, since fatty acids can serve as ligands for PPAR*β*.

PPAR*γ* has shown promise in therapy promoting terminal differentiation and apoptosis in a variety of malignancies, including liposarcoma, breast cancer, leukemia, gastric cancer, non*‐*small cell lung cancer, and prostate cancer [19–27]. In humans, the treatment of end stage prostate cancer with PPAR*γ* synthetic ligand troglitazone leads to prostate specific antigen (PSA) stabilization [150]. Although these results are promising, there are many examples of contradictory roles of PPAR*γ* in tumorigenesis. It has been observed that while PPAR*γ* agonists inhibit growth and induce apoptosis in both breast tumor cells and leukemic cells, administration of PPAR*γ* antagonists enhanced this tumor growth inhibitory effect [151, 152]. The fusion protein EWSRI/NR4A3 in extraskeletal chondrosarcomas activates PPAR*γ* expression [153]. In fibrosarcoma cells, the synthetic PPAR*γ* agonist ciglitazone induces tumor cell invasion through the generation of ROS and ERK [154].

This controversy is best exemplified by the role of PPAR*γ* in colorectal tumorigenesis. PPAR*γ* agonists have been shown to promote mouse intestinal tumors, while loss of function mutations of PPAR*γ* has been identified in human colon tumors [30, 45, 150]. Synthetic PPAR*γ* agonists promote the development of colon tumors in mice with a mutation in the tumor suppressor APC [29, 30]. This leads to increased levels of B-Catenin. Furthermore, mice diets high in saturated fats promotes tumorigenesis [155]. PPAR*γ* activation by Fas could explain the link between high fat diets and colon cancer. However, one study disputed the PPAR*γ* agonist role in tumor promotion, as it showed that PPAR*γ* agonists were able to induce differentiation and inhibit human tumors from growing in nude mice [31]. Furthermore, PPAR*γ* agonists are able to induce differentiation, cell cycle arrest, and apoptosis in human colon cancer cell lines [31]. The anti-inflammatory effects of PPAR*γ* lead to a reduced number of cancer precursor foci in inflammatory bowel disease [156]. One possible explanation for the antagonistic role of PPAR*β* and PPAR*γ* in colon tumorigenesis may be competition for RXR heterodimerization. The cellular proliferation caused by PPAR*β*, induced by specific ligands, may lead to overexpression and inhibition of PPAR*γ* heterodimerization, and subsequently contribution to human colon cancer development.

These contradictory results might be explained by species specific effects, where a combination of the different factors within each animal leads to different PPAR*γ*-associated signaling outcomes. Another example of the differences between species occurs in hepatic cancers. PPAR*γ* agonists are seen as potent carcinogens in rodents, but not seen in humans or primates [157, 158]. Furthermore, PPAR*γ* agonist rosiglitazone seems to enhance carcinogenic effects of the urinary bladder in rodents, while treatment of diabetes with pioglitazone in humans does not seem to increase the incidence of these or any other tumors [159].

While the mechanisms underlying PPAR*γ* action are not fully established, it has been shown that PPAR*γ* can inhibit the cell cycle, which is accomplished at least in part through downregulating the protein phosphatase PP2A upon PPAR*γ* activation [160]. The PPAR*γ* ligands can also inhibit the G1/S transition by inhibiting Rb phosphorylation [161]. Furthermore, PPAR*γ* upregulates the CDK inhibitors p18 and p21 [162]. PPAR*γ* ligand PGJ2 induces both CDK p21 and the proapoptotic Bax but downregulates the anti-apoptotic Bcl-xL [163]. Synthetic PPAR*γ* agonist treatment in human pancreatic cancer and bladder cancer cell lines resulted in G1 cell cycle arrest secondary to p21 induction [164, 165]. Further insight into the cross-talk between these different mechanisms will guide future antitumor therapies.

## 8. Antitumor Activity of PPAR*γ* Agonists in Osteosarcoma

Increasing evidence suggests that activation of PPAR*γ* may be explored as a possible intervention in osteosarcoma (Table 2). PPAR*γ* agonists are thought to induce terminal differentiation in adipogenesis. OS cells share many characteristics to undifferentiated osteoblasts [7–10]. Therefore, modulators that are able to promote the differentiation of these immature osteoblasts should have similar effects on the OS cells. PPAR*γ* agonist rosiglitazone has been shown to inhibit osteoblast proliferation, leading to decreased osteogenesis [88, 166]. A recent study showed PPAR*γ* to be a critical mediator underlying doxorubicin resistance in OS cell lines [167]. The chemoresistant OS cell lines were shown to have an increased expression of IL-8, which induces the antiapoptotic KLF2 [167, 168]. KLF2 is thought to negatively regulate the PPAR*γ*-induced expression of C/EBP and ADD1/SREBP, suggesting that the drug resistance may occur through the inhibition of PPAR*γ*-induced apoptosis. Furthermore, the NSAID-Associated Gene 1 (NAG-1), which is associated with NSAID-induced apoptosis, has been upregulated by PPAR*γ* in canine OS cell lines [169].

PPAR*γ* agonists and 9-cis-retinoic acid have the capability to induce osteoblastic differentiation of OS cells and inhibit OS proliferation [9, 32]. After exposure to these agents, not only did the OS cells show decreased proliferative capabilities and susceptibility to apoptosis but they also expressed increased differentiation markers, such as alkaline phosphatase. Therefore, it appears that such agents would be useful in preventing recurrence and metastasis after surgical removal of osteosarcoma. The results are further supported by the ability of PPAR*γ* to induce apoptosis in chondrosarcoma cells [177]. However, one study by Lucarelli et al. showed that treatment of human osteosarcoma cells with the PPAR*γ* agonist troglitazone promotes the *in vitro* survival via reduction in apoptosis of the malignant cells [170]. Although the mechanisms accounting for this difference are not known, it is likely that this molecular complexity results from the nuclear cross-talk and interplays between PPAR*γ* and other nuclear receptor hormones. It is plausible that the effects of PPAR*γ* agonists on OS are largely dependent on which step in the differentiation pathway the defect has occurred (Figure 2). Downstream defects may be resistant to PPAR*γ* agonists-induced terminal differentiation of its upstream counterparts. The specific factors that participate in the nuclear signaling and transcriptional regulation, along with the differentiation molecules associated with OS tumorigenesis, have yet to be fully elicited.

To the best of our knowledge, there have been no studies examining the effects of PPAR*γ* on the metastatic potential in OS. However, our notion of the potential for PPAR*γ* to reduce metastatic potential of OS is supported by examples in other tumors. Rosiglitazone has been shown to decrease the number of lung metastasis of mammary tumors in mice [178]. Dietary administration of PPAR*γ* ligands linoleic acid and conjugated linoleic acid inhibited peritoneal metastasis of colorectal tumors in nude mice [179]. Furthermore, Pioglitazone inhibited colon tumor liver metastasis in mice, possibly by downregulating Cox-2 and cyclin D1 [180]. These anti-inflammatory and other possible antiangiogenic effects of PPAR*γ* extend its potential as a chemotherapeutic agent beyond differentiation. Overexpressing PPAR*γ* in nonsmall cell lung cancer cells inhibited tumor number and metastasis [181]. Due to its ability to inhibit angiogenesis, tumor cell invasion, and inflammation, it therefore follows that PPAR*γ* would inhibit metastasis. In an analysis of primary breast tumors, PPAR*γ* expression was more often in low-grade than high-grade tumors, associated with a more favorable survival, and decreased in tumor relapses [182]. PPAR*γ* ligand troglitazone inhibited growth and liver metastasis of papillary thyroid tumors [183].

## 9. Synergistic Antitumor Activity between PPAR*γ* Agonists and Retinoids in Osteosarcoma

Receptors for retinoids include retinoic acid receptors (RARs) and retinoid X receptors (RXRs). RARs are activated by all-trans retinoic acid, a vitamin A metabolite, and heterodimerizes with RXR after ligand binding. RARs have been implicated in early embryonic morphogenesis, including development of the forebrain, hindbrain, and body axis, as well as, early signaling associated with the pancreas, heart, eye, lung, and genitourinary tracts [184–186]. Furthermore, it is able to induce the differentiation of many cancer cells and is used as an effective therapy in the treatment of acute promyelocytic leukemia by differentiating cells that express the PML-RAR*α* fusion protein [9, 32, 45, 187–189]. 

RXRs are activated by 9-cis retinoic acid. Beyond the ability of RXR to heterodimerize with many members of the nuclear receptor superfamily, such as PPARs, RXRs can form homodimers. RXRs play important roles in signaling pathways associated with development and carcinogenesis. We have recently demonstrated that exogenous expression of RAR*α* induces ligand-independent myogenic differentiation from progenitor cells [190]. We have also found that all-trans retinoic acid and 9-cis retinoid acid can effectively induce the differentiation of mouse fetal liver-derived hepatic progenitor cells [191]. RXRs are overexpressed in breast ductal carcinomas, its ablation leads to prostate and skin hyperplasia, and its overexpression sensitizes tumors to rexinoid family differentiation agents [192–195]. A synthetic rexinoid bexatrone has been developed for use in chemotherapeutic cocktails in mice mammary tumors, as well as human cutaneous T-cell lymphoma, with partial responsiveness in nonsmall cell lung cancer [196–198].

A comprehensive analysis of the possible synergistic effects between PPAR*γ* and retinoids has been carried out in a panel of OS cell lines (Table 2) [9, 32]. As a single agent, PPAR*γ* ligand troglitazone was shown to be the most effective in inducing cell death, followed by 9-cis retinoic acid [9, 32]. The strong synergistic effect on the induction of cell death was observed when both troglitazone and 9-cis retinoic acid or ciglitazone and 9-cis retinoic acid were administered to osteosarcoma cells [9, 32]. Troglitazone was shown to effectively induce alkaline phosphatase activity, a well-characterized hallmark for osteoblastic differentiation [9, 32]. These findings suggest that PPAR*γ* and/or RXR ligands may be used as efficacious adjuvant therapeutic agents for osteosarcoma as well as potential chemopreventive agents for preventing the recurrence and metastasis of osteosarcoma after the surgical removal of the primary tumors.

## 10. Other Nuclear Receptors in Osteosarcoma

Except for PPARs and retinoid receptors, several members of the nuclear receptor superfamily are also involved in the cell signaling and differentiation processes associated with OS (Table 2). Estrogens and selective estrogen receptor modulators (SERMs) are able to induce terminal differentiation in osteosarcoma cell lines through the downregulation of EGFR [173]. EGFR is a critical mediator of cell proliferation and differentiation, whose expression decreases over the course of osteoblast differentiation and maturation [199, 200]. The stimulation of estrogen receptors leads to the downregulation of EGFR in OS, resulting in cell cycle inhibition and apoptosis. Alternatively, the estrogen 17 *β*-estradiol protected osteosarcoma cells expressing estrogen receptors from etoposide-induced apoptosis [172]. However, the selective estrogen receptor modulators (SERMs) tamoxifen and raloxifene have no antiapoptosis effects.

Vitamin D receptor (VDR) has also been shown to play a role in OS cell lines and their responsiveness to therapeutic interventions. VDR is overexpressed on some OS cell lines and administration of 1, 25-dihydroxyvitamin D3 inhibits tumor growth and metastasis, while promoting terminal differentiation [174, 201]. Interestingly, it has been reported that the upregulation of VDR in OS cell lines is dependent on the tumor suppressor BRCA1 [202]. Although a synthetic VDR ligand calcitriol is able to exert antiproliferative effects in OS cells, this process is dependent on the expression of RXRs. Degradation or downregulation of RXRs causes OS resistance to the antitumor effect of calcitriol [176]. Thus, the anti-OS activity of VDR is dependent on many other nuclear proteins, including BRCA1 and RXRs.

## 11. Concluding Remarks

OS is the most frequent primary bone sarcoma, comprising approximately 20% of all bone tumors and about 5% of pediatric tumors overall. OS tumors display a broad range of genetic and molecular alterations, including the gains, losses, or arrangements of chromosomal regions, inactivation of tumor suppressor genes, and the deregulation of major signaling pathways. However, except for p53 and/or RB mutations, most alterations are not constantly detected in the majority of osteosarcoma tumors. Recent studies strongly suggest that OS may be regarded as a differentiation disease that is caused by genetic and epigenetic disruptions of osteoblast terminal differentiation. It has been well established that PPARs and retinoids play an important role in regulating osteogenic differentiation of MSCs. Increasing evidence indicates that PPAR agonists and/or retinoids can inhibit cell proliferation and induce apoptosis in cancer cells, including OS cells. PPAR agonists and/or retinoids may also inhibit OS growth by promoting osteoblastic terminal differentiation. One of the future directions is to develop safe and effective PPAR agonists and/or retinoid derivatives. These agents can be then used as adjuvant therapeutic drugs for OS therapy. Meanwhile, more thorough investigations will be needed to examine the potentially beneficial and/or adverse effects of PPAR*γ* ligands on different cells in bone and bone marrow microenvironment. Furthermore, these agents can be used as chemopreventive agents for the patients with OS who undergo the resection of the primary bone tumors in order to prevent local recurrence and/or distal pulmonary metastasis. 

## Figures and Tables

**Table 1:** Basic features of the three PPAR isoforms.

	Location	Ligands	Coactivators	Primary function	Knockout
PPAR*α*	liver, brown fat, kidney, heart, skeletal muscle	fibrates fatty acids (e.g., oleic acid, palmitic acid) eicosanoids (e.g., arachidonic acid)	p300, c/EBP, SRC-1, PBP, PGC-1, PRIP	lipid catabolism, inflammatory responses, lipoprotein metabolism	hepatomegaly, liver tumors, impaired wound healing, prolonged inflammatory responses, increased adipose tissue

PPAR*δ*	gut, kidney, brain, heart, skeletal muscle	fatty acids, NSAIDS (antagonist)	SRC-1, PBP	fatty acid *β*-oxidation, bone metabolism, tumorigenesis, vascular integrity	fatal placental defects from abnormal vasculature, small healthy adults

PPAR*γ*	adipose tissue, cartilage, osteoblasts, epithelial cells, prostate, large intestine, monocytes, kidney	thiazolinediones eicosanoids (e.g., 15d-PGJ2, 15-HETE) fatty acids (e.g., DHA, linoleic acid)	p300, c/EBP, SRC-1, PBP, PGC-1, PRIP	adipogenesis, inflammatory response, insulin sensitization, differentiation	embryo-fatal, placenta fails to implant and develop, severe metabolic, hepatic, intestinal, adipogenic, abnormalities

**Table 2:** Potential effects of nuclear receptor ligands on osteosarcoma tumors.

	Ligand	Experimental Setup	Effect	Mechanism	Reference
PPAR*γ*	Troglitazone	*in vitro* proliferation and apoptosis assays	inhibited proliferation	promotion of apoptosis, induce differentiation (alkaline phosphatase)	Haydon 2002, 2007 [9, 32]
	Ciglitazone	*in vitro* proliferation and apoptosis assays	minimal effect	unknown	Haydon2002 [32]
	Troglitazone	*in vitro* MTT proliferation and apoptosis assays	increased proliferation	inhibition of apoptosis	Lucarelli 2002 [170]
	Pioglitazone and PG J(2)	*in vitro* MTT and apoptosis assays of Chondrosarcoma Cells	inhibition of proliferation	promoted apoptosis	Nishida [171]

Retinoic Acid	9-cis retinoic acid	*in vitro* proliferation and apoptosis assays	inhibited proliferation	promotion of apoptosis	Haydon 2002, 2007 [9, 32]

Estrogen	17-beta estradiol, Ospemifene	U2OS expressing ER, *in vitro* apoptosis assays	opposed Etoposide-induced cell death	oppose increases in IL-6 and decreases in OPG, preventing osteoclast activation	Kallio 2008 [172]
	Tamoxifen, Raloxifene	U2OS expressing ER, *in vitro* apoptosis assays	no effect on Etoposide-induced cell death	unknown	Kallio 2008 [172]
	17-beta estradiol, SERMS (genistein, daidzein)	U2OS expressing ER, *in vitro* cell cycle, proliferation, apoptosis assays	inhibit proliferation, promote apoptosis	decrease EGFR, increased osteoblast maturation markers	Salvatori 2009 [173]

Vitamin D	1-alpha hydroxyvitamin D3	oral administration in Dunn murine OS model	inhibits tumor growth and metastasis	increased necrosis, no cell cycle mitotic index effects	Hara 2001 [174]
	Prolactin + 1,25 (OH)2 Vitamin D3	RT-PCR, western blots	PRL inhibits VDR expression in response to 1,25 (OH)2 Vitamin D3	VDR expression is dependent on BRCA1 expression	Deng [175]
	calcitriol	OS cells with increased RXR degradation, treated with calcitriol	increased expression of RXR restores anti-proliferative effects of calcitriol	calcitriol induced degradation is dependent on RXR expression	Prufer 2002 [176]
